# Spontaneous Pregnancy in a Hysterosalpingo-Foam-Sonography (ΗyFoSy) Cycle: A Case Report and Review of the Available Literature

**DOI:** 10.7759/cureus.37640

**Published:** 2023-04-16

**Authors:** Ioanna Lamari, Emmanouil M Xydias, Elias Tsakos, Ioannis Thanasas, Apostolos C Ziogas

**Affiliations:** 1 Faculty of Medicine, School of Health Sciences, University of Thessaly, Larissa, GRC; 2 Research Department, EmbryoClinic IVF, Thessaloniki, GRC; 3 Department of Obstetrics and Gynecology, General Hospital of Trikala, Trikala, GRC

**Keywords:** in vitro fertilization ivf, case report, fallopian tube pathology, spontaneous pregnancy, hyfosy, tubal infertility

## Abstract

Fallopian tube pathology is a very common cause of infertility for multiple couples worldwide. Tubal patency assessment is considered a crucial component of initial infertility evaluation with several evaluation tests available, such as hysterosalpingography (HSG), hysterosalpingo-contrast sonography (HyCoSy), and hysterosalpingo-foam sonography (HyFoSy), the latest tubal patency assessment, utilizing ultrasonography and a foam-based contrast agent. An additional side-benefit of these assessment tests is a fertility-enhancing effect, best studied with the application of HSG. In this report, we present a case of a 28-year-old woman with unexplained infertility who spontaneously conceived in the same menstrual cycle that the HyFoSy exam with ExEm^®^ foam (ExEm Foam Inc., Nashville, Tennessee, United States) was performed, without any additional fertility enhancement interventions.

## Introduction

Infertility is defined as the inability to conceive within 12 months of regular intercourse without contraception, with up to 12% of couples worldwide being affected by it [1]. Fallopian tube pathology is a significant contributing factor, present in over 35% of infertility cases [2]. Given the prevalence of tubal factor infertility, tubal patency assessment is an essential procedure during every basic infertility workup.

Traditionally, laparoscopy with chromopertubation was used as the main tubal patency assessment and remains the gold standard today [3]. However, newer, non-invasive techniques have taken its place in modern infertility assessment, namely X-ray hystero-salpingo-graphy (HSG) and hysterosalpingo-contrast sonography (HyCoSy) [4], with laparoscopy being reserved as a second-line assessment method in diagnostically challenging or inconclusive cases [5]. While these methods offer reliable assessment, they do come with certain disadvantages; HSG in particular, involves patient exposure to ionizing radiation and is associated with considerable intra-procedural pain and adverse reactions [6]. In 2011, hysterosalpingo-foam sonography (HyFoSy), an ultrasonographic technique that utilizes a foam-based contrast agent, ExEm® gel (ExEm Foam Inc., Nashville, Tennessee, United States), was introduced as an alternative measure demonstrating considerable imaging capabilities for tubal patency evaluation, without the disadvantages of HSG [7,8].

Despite the availability of HyFoSy and the disadvantages of more conventional methods, many fertility specialists opt for the latter, as, apart from their imaging capabilities, they have also demonstrated a positive effect on fertility, whereas the same has not been adequately demonstrated for ultrasonographic methods in the available literature [9]. In the present report, we present a case of spontaneous conception following HyFoSy examination, within the same menstrual cycle, and discuss the implications that such a report has for tubal patency assessment in the context of infertility. 

## Case presentation

A 28-year-old female, patient, gravida 0, para 0, presented to our fertility clinic with primary infertility for the past 14 months. Her BMI was within normal range, she had regular menstrual cycles and reported no dysmenorrhea or menorrhagia. She reported regular intercourse with her husband during those 14 months, without any sexual dysfunctions. From her past medical history, of note was her history of cervical lesions (cervical intraepithelial neoplasia (CIN) I, CIN II) and genital condylomas, while she had received no prior fertility treatment or undergone surgery. She was a smoker, smoking approximately 20 cigarettes per day. Her hormonal profile was within normal range, except for anti-Müllerian hormone (AMH) levels, which were low for her age. On transvaginal ultrasound (TVUS) examination, no congenital or acquired structural abnormalities were noted and she had good ovarian reserves (antral follicle count = 15). Genetic testing for thrombophilia revealed *MTHFR* double heterozygosity for C677T and A1298C mutations.

Her partner was a 32-year-old healthy man with no reported loss of libido or other intercourse dysfunctions and no urinary complaints. He reportedly smoked and consumed alcohol socially and mentioned taking protein and creatine supplements for three months in the past. Basic semen analysis revealed a borderline sperm count.

**Figure 1:**
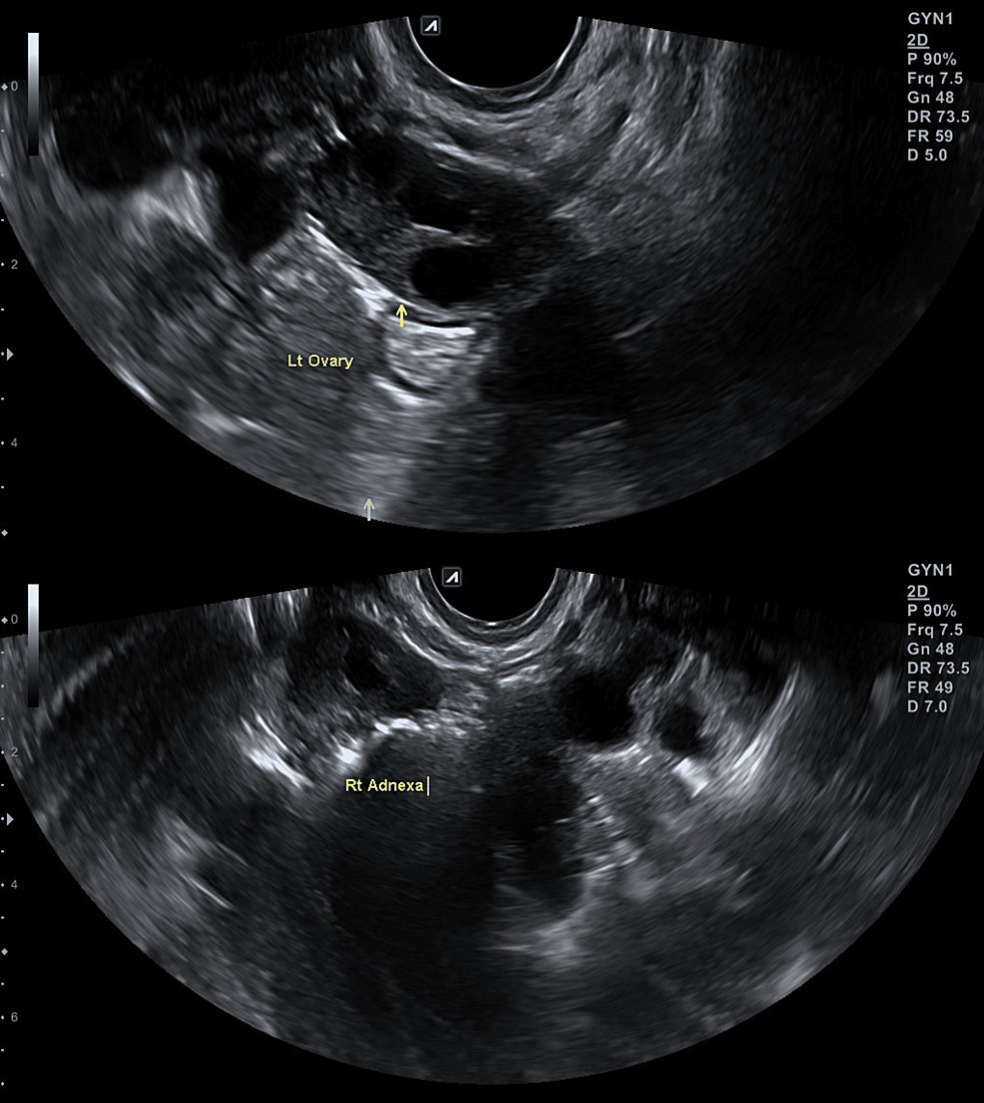
Visualization and confirmation of tubal patency bilaterally in two-dimensional images.

**Figure 2:**
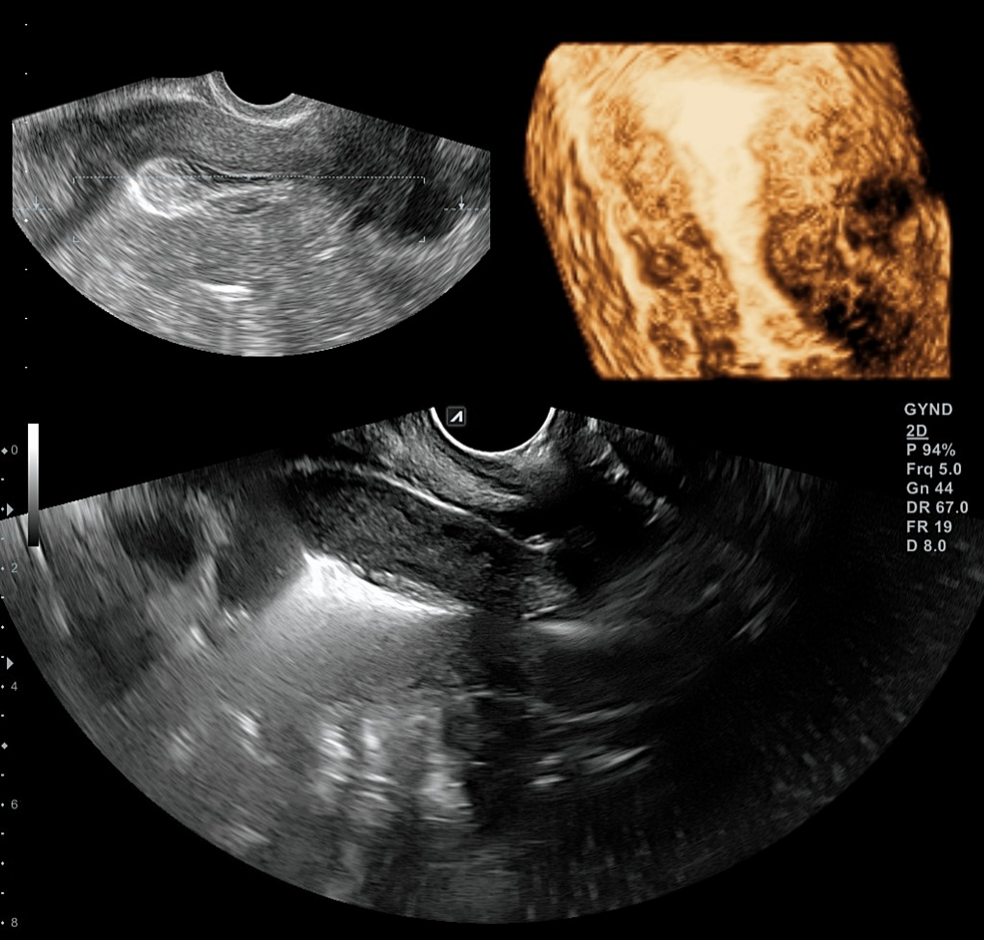
Normal uterine cavity in two-dimensional and three-dimensional images.

**Figure 3:**
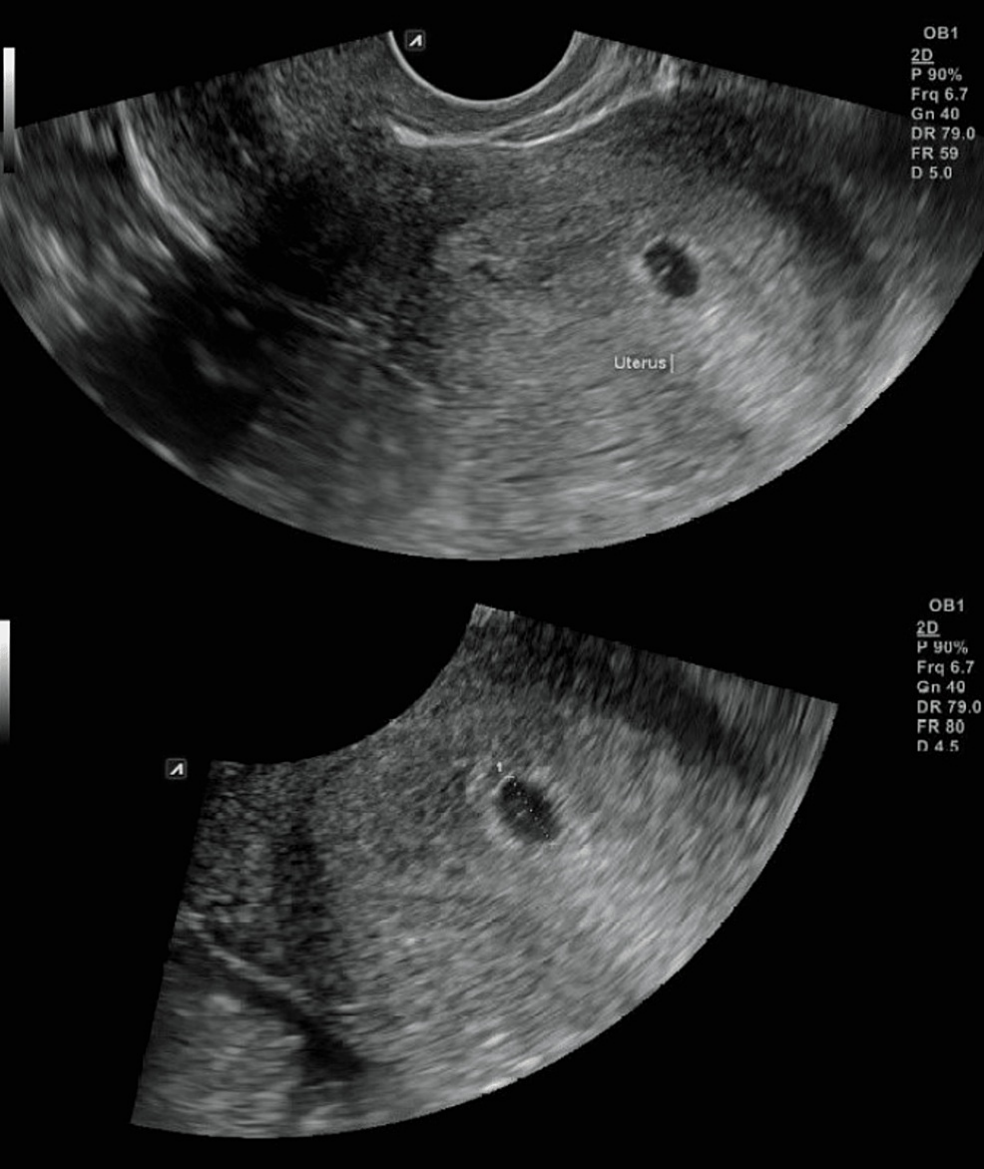
Visualization of intrauterine pregnancy sac.

Prior to any fertility treatment, we performed a HyFoSy with ExEm foam and subsequently a three-dimensional (3D) TVUS scan. The HyFoSy was conducted on the sixth day of the menstrual cycle, with low to no resistance. Both fallopian tubes were visualized and their patency was verified (Figure 1). The uterine cavity was visualized via 3D TVUS, without any abnormalities (Figure 2). Twenty-four days after HyFoSy, our patient reported a positive pregnancy test, which was later also verified ultrasonographically (Figure 3).

## Discussion

In this report, we presented the case of a young woman experiencing primary infertility for more than a year. The patient, without having received any fertility treatment, managed to achieve spontaneous conception following HyFoSy examination, within the same menstrual cycle.

Fallopian tube patency assessment is known to possess fertility-enhancing effects for certain patients, in addition to fallopian tube visualization. As early as 1966, it was suggested by Wahby et al. that HSG may enhance fertility and promote post-procedure spontaneous pregnancies [10]. They observed that one-third of patients conceived after HSG, without additional fertility interventions. They noted that younger age, shorter infertility duration, and secondary infertility were associated with an increased likelihood of spontaneous pregnancy, while nulliparity and history of abortions with decreased likelihood. Most patients conceived within six months, three of those within the same cycle as HSG, similar to our case. 

The application of HyCoSy has also been shown to have a positive impact on spontaneous pregnancy rates, like HSG, especially within the month following the examination. Bisogni et al. noted that one-third of patients with bilateral or unilateral patency had a spontaneous pregnancy 50 days following HyCoSy [11]. A prospective study by Chunyan et al. demonstrated a pregnancy rate of 19.44% within six months after HyCoSy, with a significant increase within the first 30 days after the procedure [12]. The pregnancy rate was the highest in patients with bilateral tubal patency (32.01%), which, along with the absence of injective resistance, was independently associated with a higher likelihood of pregnancy [12].

At present, HyFoSy represents a growing trend in tubal patency evaluation. It involves no exposure to radiation and minimal adverse reactions, while being less costly than HSG and performed by the fertility specialist within the clinic. Additionally, HyFoSy is reportedly less painful and better tolerated than HSG and possesses remarkable imaging capabilities [7,13]. Regarding any effects on fertility enhancement, Emanouel et al., during the first introduction of HyFoSy, demonstrated a 19% conception rate within a median of three months after the procedure [14]. Melcer et al. demonstrated a 32% pregnancy rate, with eight women conceiving within a month, similar to the present case [15]. Tanaka et al. demonstrated a 46.2% pregnancy rate within six months, with the length of infertility being a significant negative predictive factor [16]. Van Schoubroeck et al. studied 359 women post HyFoSy; 81 conceived spontaneously, with the majority of conceptions occurring within the first one to three menstrual cycles [17]. Following a similar trend, Exacoustos et al. demonstrated a 10.2% spontaneous pregnancy rate within one month after HyFoSy, 29.9% within six months, and 34.4% within 12 months [18]. The most recent study on the subject reported that one in four patients conceived naturally within 12 months after HyFoSy [19]. Although the mean time between spontaneous pregnancy and HyFoSy was four months, 18.9% of spontaneous pregnancies were noted within the first month [19], in the same month as in our case. Overall, longer infertility duration (longer than 18 months) and older age were associated with reduced conception rates [16,19].

To our knowledge, this is the first case of spontaneous pregnancy within a month after HyFoSy ever reported from Greece. Our observations are in concordance with the observed trends in the literature, namely higher spontaneous conception rates within the first months, higher rates for patients under 35 years old, and infertility duration of less than 18 months [16-19]. In our case, in addition to the aforementioned baseline characteristics, no injective resistance was noticed and patency of both tubes was visualized; both were described as positive predictors for spontaneous pregnancy [12].

## Conclusions

HyFoSy is a very promising diagnostic method for tubal patency assessment with many advantages over HyCoSy and HSG. The tubal flushing effect has already been proposed to have a positive impact on fertility patients in the past, with certain studies demonstrating a similar effect after HyFoSy as well, an observation also confirmed in the current case. Research should focus on solidifying the existence and extent of this fertility-enhancing effect, via prospective, comparative, or controlled studies, as well as investigate the risk of possible pregnancy or maternal complications after HyFoSy, in order to ensure patient safety and optimal outcomes.
